# Knowledge and practice of patients with diabetes mellitus in Lebanon: a cross-sectional study

**DOI:** 10.1186/s12889-018-5416-7

**Published:** 2018-04-20

**Authors:** Lamis R. Karaoui, Mary E. Deeb, Layal Nasser, Souheil Hallit

**Affiliations:** 1 Department of Pharmacy Practice, School of Pharmacy, Lebanese American University, P.O.Box: 36 (S23), Byblos, Lebanon; 2 Gilbert and Rose-Marie Chagoury School of Medicine, Lebanese American University, Byblos, Lebanon; 3 Department of Pharmacy, Bareen International Hospital, Abu Dhabi, United Arab Emirates; 4 Faculty of Pharmacy, Lebanese University, Hadath, Lebanon; 5 Research Department, Psychiatric Hospital of the Cross, Jal Eddib, Lebanon; 6 INSPECT-LB: Institut National de Sante Publique, Epidemiologie Clinique et Toxicologie, Faculty of Public Health, Lebanese University, Beirut, Lebanon; 7 Faculty of Pharmacy, Saint-Joseph University, Beirut, Lebanon; 8 Faculty of Medicine and Medical Sciences, Holy Spirit University, Kaslik, Lebanon

**Keywords:** Diabetes mellitus, Knowledge, Practice, Self-management, Lebanon

## Abstract

**Background:**

The objective of this study was to assess the knowledge and practice of Lebanese patients living with diabetes mellitus in regards to their diabetes self- management.

**Methods:**

A cross-sectional study, conducted between January and June 2015, enrolled 207 urban adult patients with diabetes mellitus from community pharmacies while purchasing their diabetes medications. Their knowledge and self-management practices were assessed using a structured anonymous interview survey questionnaire.

**Results:**

The mean age of the participants was 60.2 ± 15.5 years, and the Male/Female ratio was 1.38. The mean knowledge score was 2.34 ± 0.88 points (out of 6). Very few participants (17.4%) knew their current medication side effects. The mean practice score was 5.86 ± 1.77 points (out of 8). Only 15.9% of patients reported current physical activity. A multiple linear analysis showed that those with a university degree had a significantly higher knowledge (Beta = 0.448, *p* = 0.001) and practice score (Beta = 0.523 *p* = 0.047) than those with intermediate or primary schooling. Those who reported following a special diabetes diet had a higher knowledge score (Beta = 0.482, *p* < 0.001) than those who did not. Knowledge score and practice score were highly correlated (Beta = 0.844, *p* < 0.001). There was no significant differential by gender and age for knowledge and practice scores.

**Conclusions:**

The knowledge and practice scores of patients with diabetes mellitus were not satisfactory. Well-targeted interventions are needed, such as improving the communication between the pharmacist and people living with diabetes. The observed low adherence to physical exercise among patients with diabetes should also be addressed.

**Electronic supplementary material:**

The online version of this article (10.1186/s12889-018-5416-7) contains supplementary material, which is available to authorized users.

## Background

Diabetes mellitus has emerged as one of the most challenging public health problems of the twenty-first century. It is a multifaceted non-communicable disease that currently affects over 366 million people worldwide and its prevalence is likely to double by 2030 [1, 2]. Type 2 diabetes mellitus (T2DM) among adults in Lebanon is estimated to be approximately 15.8%, similar to what is observed in industrialized countries, but lower than other countries in the region, namely Bahrain (25.5%), United Arab Emirates (UAE) (23.3%) and Kingdom of Saudi Arabia (KSA) (23.7%) [3, 4]. The increase in prevalence and incidence of diabetes mellitus has been attributed to changes in lifestyle risk behaviors due to urbanization associated with sedentary lifestyles and unhealthy diets. The social and economic burden of patients living with diabetes on the health care system is substantial, due to high treatment expenditures, lost productivity and economic growth [5]. Diabetes mellitus leads to many complications that are associated with higher morbidity and mortality, when not properly controlled [6, 7]. These complications can affect the microvascular (neuropathy, nephropathy and retinopathy) or macrovascular (cardiovascular or peripheral vascular disease) systems and are unfortunately, irreversible once they occur [7]. The American Diabetes Association (ADA) and the International Diabetes Federation (IDF) both advocate self-management to be a core component of diabetes care [8]. Good T2DM control is often difficult to achieve due to the required lifestyle changes, including eating habits, body weight, exercise and self-monitoring of blood glucose [9]. The adequate counseling of patients with diabetes mellitus is still suboptimal during the physician patient encounter due to time constraints and the lack of mentioning and discussing patients’ self-care motivation [10]. Studies that examine diabetes knowledge and practice are limited in the Middle East North Africa (MENA) region and especially in Lebanon. Lebanon is a small, middle-income country in the Middle East experiencing the third stage of its demographic transition where fertility and mortality rates have both declined [11]. The majority of the Lebanese population lives in urban areas with increasing levels of physical inactivity and obesity rates [12]. The present study aims at assessing the knowledge and practice of Lebanese patients living with diabetes mellitus, in regards to diabetes self-management including current management practices and disease monitoring. Understanding the present knowledge and practices of patients with diabetes mellitus is a cornerstone to plan well-targeted interventions in order to improve and alleviate the burden of diabetes care.

## Methods

### Study population

A cross-sectional study, conducted between January and June 2015, gathered data from 207 adult patients aged 18 years old and above living with diabetes mellitus based on validated surveys adapted to the Lebanese context. It was undertaken in six community pharmacies in Beirut and Mount Lebanon governorates that served as a practice training sites for fifth year Lebanese American University pharmacy students over 2 months. All patients above 18 years of age, with diabetes mellitus presenting to the pharmacy to get their prescribed medications, were approached by the student trainee for potential enrollment in the study. After giving a verbal consent to participate, the patient was interviewed. Excluded were patients below 18 years of age and patients with dementia or any cognitive function decline. If an individual was bringing medications on behalf of a patient with diabetes mellitus, this individual was not interviewed for consistency and accuracy of information to avoid any information bias.

To ensure consistency in the data collection process, all students underwent a training session on survey administration. A sample of 204 patients was targeted to allow for adequate power for bivariate and multivariate analyses to be carried out based on a population size of four million inhabitants in Lebanon, a 15.8% expected frequency of diabetes [3, 4] and a 5% confidence limits [13]. The non- response rate was (18.0%) 250 patients were approached, 207 agreed to participate.

The study was reviewed and approved by the Lebanese American University Institutional Review Board (IRB) ethical committee. Verbal consent was obtained rather than written consent, given that agreeing to participate in the interview survey study was considered by the IRB equivalent to a formal consent.

### Questionnaire

A pilot study on twenty individuals was undertaken in order to validate the survey instrument and train the interviewers. The questionnaire gathered data on the participants’ socio-demographic characteristics, including age, gender, region, diabetes family history and educational level, defined as illiterate, primary level (less than 8 years of education), complementary (more than 8 years), high school (completed high school) and university (having a university degree). Data on when and how they were diagnosed with diabetes mellitus, reported perceived co-morbidities, current medication history and disease complications.

To assess the patients’ knowledge about the disease, patients were asked if they knew their glucose and glycated hemoglobin (HbA1c) levels and the normal ones, how often they underwent an eye and foot exams, the weekly time of exercise and their knowledge about the complications of diabetes, how to self-administer insulin injections, the possible side effects from the treatment. (Additional file 1).

### Score measurement

Patients’ knowledge score of diabetes was assessed by six questions whether the patients 1) knew the name of the diabetes medication they were taking 2) and its side effects, 3) the desired normal fasting blood glucose levels and 4) the target HbA1c levels, to achieve glycemic control 5) whether diabetes is not a contagious disease and 6) what type of foods should a patient with diabetes mellitus avoid. Following the ADA’s recommended guidelines, target HbA1c levels defined as good glycemic control for HbA1c less than 6.5%. [14] Answer choices were given a numerical value of 1 for having correct knowledge and 0 for lack of knowledge. The total knowledge score ranged between 0 reflecting low/lack of knowledge and 6 reflecting correct/high knowledge.

Patients’ practice score was assessed using 8 questions related to following a special diet and to physical activity. Physical activity was self-reported by the patient, and measured by the duration that the patient spends on physical exercise per week, with 0 points for not exercising, 1 point for exercising less than 30 min per week, 2 points for exercising between 1 and 3 h and 3 points for exercising more than 3 h. Four questions were related to diabetes monitoring such as self-monitoring of blood glucose, having had a creatinine test, routine eye exam and whether the patient reports taking a sugary food or drink immediately when having a hypoglycemic episode. The frequency of blood sugar self-testing was scored as follows: 0 for no self-testing, 1 for less than once daily self-testing, 2 for once daily self-testing, 3 for 2–3 times self-testing per day, and 4 for more than 4 times self-testing per day. The practice variable is the sum of answers on these 8 questions. Answer choices for six questions were given a numerical value of 1 for positive practice and 0 for negative practice. The total score ranged from 0 as to no practice, to 13 reflecting a full positive practice.

### Statistical analysis

All analyses were conducted using Statistical Package for the Social Sciences (SPSS) v. 23. Statistical significance was set at *p*-value less than 0.05. Student’s t-test was used to assess differences in means of continuous variables. Differences in proportions of qualitative variables by gender were calculated using Chi-square test. The analysis of variance (ANOVA) test was used to compare multiple group means. For the multivariate analysis, a linear regression was performed, in order to assess the independent effect of co-variates on the knowledge and practice scores. Multivariate analysis linear regressions were carried out taking the knowledge and practice scores as dependent variables respectively, and using variables that showed a *p* < 0.2 in the bivariate analysis [15, 16]; potential confounders may be eliminated only if *p* > 0.2, in order to protect against residual confounding [17].

## Results

### Sample characteristics

The sample consisted of 58% males and 42% females. The mean age for the total sample was 60.29 ± 14.04 years with no gender difference in mean age distribution. Forty-five percent had a primary level education and 24.2% completed high school and the remaining 30.4% had a university degree. More than half of the sample were current smokers, with males significantly smoking more than females (55% vs 32.2%; *p* = 0.001), with a mean daily number of cigarette smoking of 15.45 ± 12.95. In addition, there was no gender differential for hubble-bubble smoking (*p* = 0.183) (Table 1).

### Clinical characteristics of the patients

Table 2 shows that 94% of the sample had T2DM and only 12 patients had T1DM. A positive family history of diabetes mellitus was reported by 76.8% of the sample with no sex differential. Around 60% of the sample with T2DM had also reported concurrent chronic conditions such as hypercholesterolemia, hypertension, cardiovascular disease, and pulmonary disease. Males with T2DM were significantly more likely (65.0%) to report other comorbidities than females (51.7%) (*p* = 0.006). The diagnosis of diabetes mellitus was reported by coincidence among 40% of the surveyed population with no significant sex differential. When asked about how long they have been diagnosed with diabetes, 45.2% of patients reported the duration to be within the last 5 years, 29.1% since 6–10 years and 25.7% since 11 years and more. The majority of patients (89.4%) were taking oral hypoglycemic agents, while 16% were on insulin therapy with no sex differential for both (*p* = 0.423 and *p* = 0.229 respectively). The oral hypoglycemic agents that patients were taking belong to the following pharmacologic classes: dipeptidyl peptidase IV inhibitors (4.8%, *n* = 10), biguanides (59.4%, *n* = 123), sulfonylureas (26.6%, *n* = 55), meglitinide derivatives (2.4%, n = 5), thiazolidinediones (1%, *n* = 2) and 15.9% (*n* = 33) of patients were receiving a combination of two or more oral agents.

**Table 1:** Distribution of selected characteristics of patients with diabetes mellitus by gender

	Total		Male		Female		*P*-value
	N	(%)	N	(%)	N	(%)	
	207	100.0	120	57.9	87	42.1	
Age group (years)							0.02
< 20	3	(1.4)	0	(0.0)	3	(3.4)	
20–29	2	(1.0)	0	(0.0)	2	(2.3)	
30–39	9	(4.3)	7	(5.8)	2	(2.3)	
40–49	27	(13.0)	20	(16.7)	7	(8.0)	
50–59	48	(23.2)	23	(19.2)	25	(28.7)	
60+	118	(57.0)	70	(58.3)	48	(55.2)	
Mean ± SD	60.3 ± 14.0		60.4 ± 13.0		60.2 ± 15.5		0.93
Educational level							0.16
Primary/intermediate	94	(45.4)	51	(42.5)	43	(49.4)	
High school	50	(24.2)	26	(21.7)	24	(27.6)	
University	63	(30.4)	43	(35.8)	20	(23.0)	
Current smoker (any)	118	(57.0)	75	(62.5)	43	(49.4)	0.06
Cigarette smoker	94	(45.4)	66	(55.0)	28	(32.2)	0.001
Hubble-bubble smoker	28	(13.5)	13	(10.8)	15	(17.2)	0.18
Physical Activity							0.148
None	134	(64.7)	72	(60.0)	62	(71.3)	
Less than 30 min/week	40	(19.3)	29	(24.2)	11	(12.6)	
1–3 h/week	26	(12.6)	16	(13.3)	10	(11.5)	
More than 3 h/week	7	(3.4)	3	(2.5)	4	(4.6)

**Table 2:** Clinical characteristics of patients

	Total		Male		Female		*P*-value
	N	(%)	N	(%)	N	(%)	
Current chronic illness							0.006
Type 1 diabetes only	8	(3.9)	1	(0.8)	7	(8.0)	
Type 1 diabetes + other^a^	4	(1.9)	4	(3.3)	0	(0.0)	
Type 2 diabetes only	72	(34.8)	37	(30.8)	35	(40.2)	
Type 2 diabetes + other^a^	123	(59.4)	78	(65.0)	45	(51.7)	
Family history of diabetes	159	(76.8)	91	(75.8)	68	(78.2)	0.70
Method of diagnosis							0.26
Coincidence	83	(40.1)	52	(43.3)	31	(35.6)	
Physician	124	(59.9)	68	(56.7)	56	(64.4)	
Taking diabetes medications	195	(94.2)	114	(95.0)	81	(93.1)	0.56
Taking insulin	33	(15.9)	16	(13.3)	17	(19.5)	0.23
Other concurrent medications							
Aspirin	98	(47.3)	63	(52.5)	35	(40.2)	0.08
Lipid lowering	87	(42.0)	51	(42.5)	36	(41.4)	0.87
Side effects from medications	36	(17.4)	20	(16.7)	16	(18.4)	0.75
Duration of diagnosis (years)							0.27
< 3	44	(21.4)	28	(23.5)	16	(18.4)	
3–5	49	(23.8)	23	(19.3)	26	(29.9)	
6–10	60	(29.1)	34	(28.6)	26	(29.9)	
11+	53	(25.7)	34	(28.6)	19	(21.8)	

^a^Other chronic conditions include: high cholesterol, heart disease, high blood pressure, lung disease

The participants were also asked about the type of concurrent intake of aspirin and/or lipid lowering agent. The proportion of patients receiving aspirin and lipid lowering agents were 47.3% and 42% respectively. However, males were more likely to be taking aspirin (52.5%) compared to 40.2% among females with a borderline significance (*p* = 0.08) (Table 2).

### Reported complications

Table 3 shows the short-term complications reported by patients; hypoglycemic episodes were reported by 30% of patients while hyperglycemic episodes were reported by 50.7% with no sex differential for both variables as well (*p* = 0.219 and *p* = 0.698 respectively). The most common long-term complications were blurry vision (36.7%) and neuropathy (35.3%), followed by proteinuria (15.5%), foot ulcers (14%), and loss of sensation (9.2%), while renal problems were the least frequent. However, blurry vision (*p* = 0.04) and proteinuria (*p* = 0.01) were the only two complications that were significantly more likely to be reported by males compared to females. Patients who were taking insulin were asked if they know how to self-administer insulin injection. Out of 33 patients on insulin therapy, 20 (60.6%) reported self-administration of the insulin injection.

**Table 3:** Complications reported by patients due to diabetes mellitus

	Total		Male		Female		*P*-value
	N	(%)	N	(%)	N	(%)	
Short-term complications							
Hypoglycemic episode	62	(30.2)	32	(26.9)	30	(34.9)	0.22
Hyperglycemic episode	104	(50.7)	59	(49.6)	45	(52.3)	0.70
Long-term complications							
Blurry vision	76	(36.7)	51	(42.5)	25	(28.7)	0.04
Proteinuria	32	(15.5)	25	(20.8)	7	(8.0)	0.01
Renal problems	9	(4.3)	6	(5.0)	3	(3.4)	0.59
Neuropathy	73	(35.3)	37	(30.8)	36	(41.4)	0.12
Loss of sensation	19	(9.2)	10	(8.3)	9	(10.3)	0.62
Foot ulcers	29	(14.0)	18	(15.0)	11	(12.6)	0.63

### Knowledge score

Most of the patients recognized the name of the diabetes medication (94.2%) they were currently taking only 17.4% knew their side effects. The proportion that identified correctly the HbA1c and glucose level to maintain proper diabetes control, were 57% and 78.7% respectively. More than three quarter (87.4%) knew which foods patients with diabetes mellitus should avoid eating, 88.4% reported that diabetes was not contagious. The mean knowledge score was 2.34 ± 0.88 points, with the patient’s level of education and smoking status being significantly associated with the diabetes knowledge score (*p* = 0.011 and *p* = 0.033 respectively).

### Practice score

The reported positive practices regarding the 8 questions used to construct the practice score were also evaluated. The lowest positive practice score was current prevalence of physical activity (15.9%). Questions related to participants’ practices to control and manage their diabetes were as follows: 15.9% were on insulin therapy, and 89.4% with oral hypoglycemic agents, 59.9% were using also a special diet therapy. When asked about measures taken to monitor their disease, 66.7% of patients performed serum creatinine testing, and 80.7% had an eye exam. The use of a self-monitoring glucose device was reported by 71.5% of patients. The majority (96.1%) did manage adequately a hypoglycemic episode. Table 4 shows the mean practice score was 5.86 ± 1.77 points, with no differential between males and females (*p* = 0.484). Age (*p* = 0.032) and the level of education (*p* = 0.001) were significantly associated with the practice score. It is worth noting that the knowledge and practice scores were significantly correlated (*p* < 0.001; *r* = 0.433).

**Table 4:** Bivariate analysis of the knowledge and practice scores by selected characteristics

	Knowledge			Practice		
	N	Mean ± SD	*p*-value	N	Mean ± SD	*P*-value
Gender			0.611			0.455
Male	120	2.37 ± 0.86		119	5.78 ± 1.57	
Female	87	2.31 ± 0.93		86	5.97 ± 2.01	
Educational level			0.011			0.001
Intermediate/Primary	93	2.15 ± 0.92		93	5.37 ± 1.68	
High school	50	2.36 ± 0.82		49	6.12 ± 1.81	
University	63	2.61 ± 0.83		62	6.33 ± 1.68	
Diabetes Family history			0.645			0.821
No	48	2.29 ± 0.98		47	5.91 ± 2.18	
Yes	159	2.36 ± 0.85		158	5.84 ± 1.63	
Smoking			0.033			0.119
Never smoker	113	2.23 ± 0.95		112	6.03 ± 1.93	
Ever smoker	94	2.48 ± 0.78		93	5.65 ± 1.53	
Physical activity level			0.177			
Non-active	134	2.31 ± 0.87				
Moderate activity	40	2.25 ± 0.95				
Heavy activity	33	2.60 ± 0.82				
Age	*r* = −0.057		0.415	*r* = −0.15		0.032

### Multivariate linear regression analysis

The results of the multivariate analyses of the independent association of the co-variates age, sex, educational level, smoking, physical activity and following a special diet on the knowledge score are presented in Table 5. The patients with a university degree had a higher knowledge score (Beta = 0.448, *p =* 0.001) than those with a lower educational level. The other co-variate significantly correlated with an increased diabetes-related knowledge (Beta = 0.482, *p* < 0.001) was among those following a special diet. Table 5 shows also the independent association of the co-variates age, sex, educational level, smoking, physical activity and knowledge score on the practice score. The linear regression of the practice score as a dependent variable showed that knowledge score would significantly increase the practice score (Beta = 0.844, *p* < 0.001). Furthermore, having completed high school (Beta = 0.322 *p* = 0.02) and a university educational level (Beta = 0.523 *p* = 0.047) would also significantly increase the practice score.

**Table 5:** Multiple linear regression of practice and knowledge score by selected variables among patients with diabetes

	Practice score			Knowledge score		
	Unstandardized Beta	Standardized Beta	*p*-value	Unstandardized Beta	Standardized Beta	*p*-value
Age	−.013	−.103	.094	−.004	−.070	.308
Gender ^a^	.204	.057	.362	−.035	−.019	.774
Smoking ^b^	.676	.189	.003	−.234	−.130	.053
Knowledge score	.844	.425	< 0.001			
University level of education ^c^	.523	.136	.047	.448	.232	.001
High school level of education ^c^	.322	.156	.020	.066	.064	.372
Diet ^d^				.482	.266	.000
Heavy activity ^e^				.134	.100	.148
Moderate activity^e^				−.153	−.068	.322

^a^reference group: male gender

^b^reference group: never -smokers

^c^reference group: primary/ intermediate level of education

^d^reference group: no special diet

^e^reference group: non active

## Discussion

The study results revealed that a university level of education and following a special diet were significantly associated with an increase in the knowledge score, whereas the knowledge score, a high school and a university level of education were significantly associated with an increase in the practice score.

In several studies, age was found to negatively correlate with the knowledge level about diabetes; younger persons tended to be more informed than their older mates [18, 19]. No significant association was found between age and diabetes-related knowledge in our study, however our results indicated that patient’s age was negatively and significantly associated with a higher practice score. An alternative explanation of such results may relate to the small sample enrolled in this study. Indeed, our findings confirm that younger patients have a better understanding of the disease, thus better practices. There was no significant sex differential regarding the mean knowledge score. One study in the UAE found that males had a higher mean knowledge score than females which is not consistent with other studies in other contexts [20]. Studies from both developed and developing countries have reported that diabetes-related knowledge is generally poor among patients with diabetes mellitus [21–24], in line with our study results that showed that knowledge and practice scores of diabetes mellitus management and monitoring were also below average. A recent study in Kuwait revealed a considerable diabetes knowledge gap with only 6% of participants demonstrating high levels of diabetes-related knowledge [25]. Most of the respondents in our study, knew the drug name of the diabetes medication they were prescribed, however barely 20% were aware of its side effects. Previous studies on patients with diabetes mellitus reported limited knowledge of diabetes medication, of the drug’s mechanism of action and associated side effects in particular [26]. Given the burden of polypharmacy with diabetes, patients are on multidrug regimens, which may explain why they would not be able to recognize the potential side effects of every medication they are taking including those for diabetes. Studies have demonstrated that diabetes-related knowledge is limited, with most suggesting educational programs to increase patient awareness about diabetes mellitus to improve their ability to cope with the disease [20, 27–32]. Nazir et al. reported a significant but weak positive correlation between diabetes-related knowledge and medication adherence in outpatients with diabetes mellitus in Pakistan (*r* = 0.036, *p* < 0.05) [33]. Furthermore, knowledge and practice of diabetes were significantly correlated, demonstrating that an increased diabetes-related knowledge would improve the patient’s practice leading to a better control and management of the disease. Educational level of the participants was significantly correlated with a higher knowledge and practice score. A patient with diabetes mellitus who completed high school or university studies had a significantly increased knowledge and practice score compared to those who completed primary or complementary school. Our results concur with previous studies [20, 34–37] and with a recent systematic review [38]. Thus, patient education about the etiology of diabetes should be addressed and clarified by healthcare professionals, along with the associated complications of the disease. This can be achieved by encouraging health education in schools and by using the appropriate language and communication tool to educate the public on diabetes. Education should therefore not be limited to clinics and patients with diabetes mellitus only. The emergence of structured patient education facilitates successful diabetes self-management, and improves patient’s adherence and compliance to appropriate pharmacotherapy [39]. The future is to test whether these approaches, if adapted to the Lebanese context, will be beneficial and culturally-accepted. Diet was shown to increase the practice score in patients with diabetes enrolled in this study. Diet plays an important role in the prevention and management of diabetes mellitus. Diet is extremely challenging as it is an everlasting choice that patients with diabetes mellitus face every day their desire to eat the food they know they are not supposed to have. This conflict might be a major component in understanding the participants’ low rates of positive practices for handling diabetes, results similar to those of Saleh et al. [40].

Physical exercise was barely reported by 16% of the sample. Given the importance of exercise in the management of diabetes, it should be specifically addressed. A previous study showed that the prevalence of T2DM in Lebanon significantly increased with increasing body mass index [41]. A population-based study showed that 10% of Lebanese adults with diabetes were physically active [42]. Research demonstrated that patients are more likely to engage in exercise when recommended by their healthcare professionals as part of diabetes management. Patient counseling about the importance of exercise in diabetes management should definitely be implemented by Lebanese physicians and pharmacists.

With the increased cost of living in Lebanon, and the restrictions of healthcare plans, patient counseling and diabetes education provided in community pharmacies, by licensed pharmacists, constitute one of many and probably the most efficient interventions. Patients may consult and seek advice from community pharmacists without prior appointment unlike other health professionals. Pharmacists usually have long-term fiducial relationships with most of their chronic patients including those with diabetes. They do not only dispense medications, but play a dynamic role in quality diabetes care through detection, referral of individuals at risk of diabetes and education of patients about their medications’ dosage, side effects, administration and monitoring parameters [43, 44]. Pharmacist should be specifically trained to communicate with patients, and empower them to self-manage their disease through knowledge transfer and counseling on lifestyle changes and drug therapy.

### Limitations

This study is cross-sectional and comprises a select sample of patients living with diabetes, who were presenting solely to private pharmacies in urban areas. The sample may not be representative of all people living with diabetes in Lebanon since the sample was taken from two governorates only. Our findings therefore may reflect an overestimate of the level of knowledge and practice of diabetes mellitus patients in Lebanon. We reviewed validated questionnaires but opted to create a shorter one, given the context in which we were recruiting patients for this study, in order to capture medication-related patient details, in addition to assessing knowledge and practice of diabetes. The questionnaire relied on patients’ self-rated assessment of their own practice and therefore patients might have felt pressured into completing the questionnaire or might have been unwilling to reveal their practice deficiencies. Furthermore, the use of a questionnaire in patients may not always be accurate: problems in question understanding, recall deficiency and over or under evaluating symptoms may still exist, which may lead to a possible information bias.

## Conclusions

Based on our findings, Lebanese patients with diabetes were shown to have poor knowledge and practice about their disease. The development of health literacy, patient counseling and education programs are much needed in the community pharmacy setting. Further larger studies are warranted to evaluate knowledge, attitude and practice among patients with diabetes in Lebanon.

## Additional files

Additional file 1: Knowledge and Practice of Diabetes Mellitus in Lebanon: A Cross-Sectional Study – Interview Survey. (PDF 241 kb)

## Abbreviations

ADA: American diabetes association
ANOVA: Analysis of variance
HbA1c: Glycated hemoglobin
IDF: International diabetes federation
IRB: Institutional Review Board
KSA: Kingdom of Saudi Arabia
MENA: Middle East and North Africa
SPSS: Statistical package for the social sciences
T1DM: Type 1 diabetes mellitus
T2DM: Type 2 diabetes mellitus
UAE: United Arab Emirates
