# Supplementary material for: Knowledge and practice of patients with diabetes mellitus in Lebanon: a cross-sectional study
Source: BMC Public Health. 2018 Apr 20;18:525. doi: 10.1186/s12889-018-5416-7 (PMC5910622; doi:10.1186/s12889-018-5416-7)
Supplement: Supplementary file 1 — Knowledge and Practice of Diabetes Mellitus in Lebanon: A Cross-Sectional Study – Interview Survey. (PDF 241 kb) [file 12889_2018_5416_MOESM1_ESM.pdf]

Knowledge and Practice of Diabetes Mellitus in Lebanon: A Cross-Sectional Study

GENERAL INFORMATION

G1. Age (years):___________________________
G2. Weight (Kg): _________________________
G3. Gender: _____________________________
a. Male
b. Female

G4. Education
a. Primary/Intermediate
b. High school
c. University

G5. Smoking
a. Cigarettes (#/day: _____________)
b. Hubble bubble (#/day: _________)
c. No

G6. Family history of diabetes
a. Yes
b. No

G7. Please indicate which chronic condition you have (Check all that apply):
a. Type 1diabetes mellitus
b. Type 2 diabetes mellitus
c. High cholesterol
d. High blood pressure
e. Heart disease
f. Lung disease

G8. Are you on Aspirin?
a. Yes
b. No

G9. Are you on lipid-lowering medications (statins, fibrates..)?
a. Yes
b. No

G10. Do you think diabetes mellitus is contagious?
a. Yes
b. No
c. Don't know

DIAGNOSIS AND MONITORING

D1. How did you know you had diabetes?
a. Coincidence/curiosity
b. Physician check-up

D2. Do you have a machine to measure your blood sugar level?
a. Yes
b. No

D3. How often do you test your own blood glucose levels? (Please tick one box only)
a. >4 times/day
b. 2-3 times/day
c. Once a day
d. < 1time/day
e. Never

D4. Do you know what is the normal fasting blood glucose level?
a. Yes. Specify:_______
b. No
c. Don't know

D5. Do you know your HbA_1c level?
a. Yes. Specify:_______
b. No
c. Don't know

D6. Do you know normal HbA_1c level
a. Yes. Specify:_______
b. No
c. Don't know

D7. When were you first diagnosed? ___________________________

D8. How often do you have a foot exam per year? _______________

D9. How often do you have an eye exam per year? _______________

MANAGEMENT – NON-PHARMACOLOGICAL THERAPY

MNP 1. Do you follow a special diet for diabetes?

a. Yes
b. No

MNP 2. Do you know what food you need to avoid if you have diabetes mellitus?

a. Yes. Specify.______________________
b. No
c. Don't know

MNP3. How much exercise are you doing to help manage your diabetes per week?

a. <30 minutes
b. 1- 3 hours
c. > 3 hours
d. I am not doing any exercise

MANAGEMENT – PHARMACOLOGICAL THERAPY

MP1. How do you currently control your diabetes? (Please tick all that apply)

a. Insulin
b. Oral tablets
c. Diet
d. Physical activity
e. Other. Specify:_________________________

MP2. Do you know the name of your medication (s)?

a. Yes. Specify __________________________
b. No

MP3. Do you know what side effects you may have from your medication (s)?

a. Yes. Specify___________________________
b. No

MP4. Are you on insulin?

a. No
b. Yes. Please tick below:
   i. *Short acting:*
      a. Actrapid ®
      b. Humalog®
      c. Novorapid®
      d. Humilin R®
   ii. *Long acting:*
      a. Levemir®
      b. Lantus®
   iii. *Intermediate acting:*
      a. Humalog 25/75®
      b. Humalog 50/50 ®
      c. Mixtard®
      d. Humilin N ®
      e. Humilin 70/30®
      f. Novomix®

MP5. How many insulin shots/day? __________________

MP6. Do you know how to self-administer insulin?

a. Yes
b. No

COMPLICATIONS

C1. Have you ever had a hypoglycemic episode?

a. Yes
b. No

C2. Have you ever had a hyperglycemic episode?

a. Yes
b. No

C3. Do you respond to a low blood sugar by taking a sugary food or drink immediately?

a. Yes
b. No, please state what you do________________

C4. Have you ever seen an eye doctor?

a. Yes
b. No

C5. Did you have blurry vision or decreased visual acuity?

a. Yes
b. No

C6. Have you ever had protein in urine?

a. Yes
b. No

C7. Did you ever check your blood creatinine levels?

a. Yes
b. No

C8. Did you ever require dialysis/kidney transplant?

a. Yes
b. No

C9. Have you ever had any numbness or tingling or burning of fingers or toes?

a. Yes
b. No

C10. Have you ever had any loss of sensation to a body part or area?

a. Yes
b. No

C11. Have you ever had foot ulcers or foot deformities?

a. Yes
b. No
